# Influence of Dietary Supplementation with *Boswellia serrata* and *Salix alba* on Performance and Blood Biochemistry in Free-Range Leghorn Laying Hens

**DOI:** 10.3390/vetsci9040182

**Published:** 2022-04-11

**Authors:** Alessandro Guerrini, Thomas Dalmonte, Caterina Lupini, Giulia Andreani, Roberta Salaroli, Giulia Quaglia, Augusta Zannoni, Maurizio Scozzoli, Monica Forni, Gloria Isani

**Affiliations:** 1 Department of Veterinary Medical Sciences, University of Bologna, Via Tolara di Sopra 50, Ozzano dell’Emilia, 40064 Bologna, Italy; alessandro.guerrini5@unibo.it (A.G.); caterina.lupini@unibo.it (C.L.); giulia.andreani2@unibo.it (G.A.); roberta.salaroli@unibo.it (R.S.); giulia.quaglia2@unibo.it (G.Q.); augusta.zannoni@unibo.it (A.Z.); monica.forni@unibo.it (M.F.); gloria.isani@unibo.it (G.I.); 2 Interdepartmental Centre for Agri-food Industrial Research (CIRI Agrifood), University of Bologna, Piazza G. Goidanich 60, 47521 Cesena, Italy; 3 Health Sciences and Technologies Interdepartmental Center for Industrial Research (CIRI-SDV), University of Bologna, 40126 Bologna, Italy; 4 Independent Researcher, 47521 Forlì, Italy; maurizio@apabio.it

**Keywords:** phytoextracts, Leghorn hen, *Boswellia serrata*, *Salix alba*, free-range

## Abstract

This study was conducted to evaluate the safety and the beneficial effects of dietary supplementation with *Boswellia serrata* (*Bs*) and *Salix alba* (*Sa*) in Leghorn hens during the critical pre-laying and laying phases. A total of 120 pullets, 17 weeks of age, were assigned to two groups (Control—C; Treated—T, *n* = 60 each). For 12 weeks, the T group received a diet supplemented with 0.3% of dry extracts of *Bs* (5%) and *Sa* (5%). The study lasted 19 weeks. Productive performance, serum analytes, H/L ratio, IgA and anti-IBV antibodies were investigated. Water intake was significantly higher, while body and egg weight was significantly lower for the T group (*p* < 0.05). No other differences were detected in performance parameters, serum analytes, IgA and H/L ratio excluding t0, with a significantly (*p* < 0.05) higher H/R ratio and higher titers of anti-IBV antibody for the T group. Overall, the data obtained in this study show that the supplementation with *Bs* and *Sa* was safe and resulted in an increase in water consumption, a decrease in egg weight, and a sedative effect in the hens. In the future, it would be interesting to test this supplement in hens reared on intensive farms.

## 1. Introduction

In recent years, attention to nutrition-based health strategies and the interest in alternative approaches to reduce antibiotic use in animal production have grown [1,2]. In fact, organic farming, in particular for laying hens, represents an attractive perspective due to the increasing environmental awareness worldwide and the increasing consumer attention to animal welfare [2]. Antibiotic growth promoters (AGP) are useful in enhancing the growth performance and improving the feed efficiency in livestock, including poultry, while at the same time reducing intestinal inflammation due to productive stress [3]. However, the excessive use of AGP in animal diets enhances the development of antibiotic-resistant bacterial strains, which in turn determines negative effects on animal and human health, including dysbiosis [4]. In this regard, phytoextracts are considered an attractive alternative option. Phytoextracts are widely used in modern poultry production to improve welfare and productivity [5]. They include, for example, feed additives also approved for use in poultry production, such as *Boswellia serrata* and *Salix alba* extracts [6].

The resin of *B. serrata* (*Bs*) (Fam. *Burseraceae*), also called Indian olibanum, is obtained from trees native to India and is widely used for the treatment of inflammatory diseases in animals, including those affecting the gastrointestinal tract (e.g., diarrhoea, dysentery, and inflammatory bowel disease), due to the bioactive compounds contained therein. Particularly, boswellic acids pentacyclic triterpenes with 3-acetyl-11-keto-β-boswellic acid (AKBA), 11-keto-β-boswellic acid (KBA) and β-boswellic acid (BA) characterised by the highest biological activities. These compounds are responsible for anti-inflammatory, antiseptic, analgesic, antibacterial, anticancer, hepatoprotective, hypolipidemic, hypocholesterolemic, immunomodulatory, and antiproliferative effects in vitro and in vivo [7,8,9,10,11,12,13]. The supplementation of this phytoextract in broiler diets improves the breeding performance and the digestibility of nutrients due to the microbiological stabilisation of the small intestine by the activity of boswellic acids [14,15]. Moreover, some studies have reported the hypolipidemic effects of *Bs* and an improvement in the quality of poultry meat [16,17,18].

The bark of *S. alba* (*Sa*), known as white willow, has been used since ancient times in folk medicine for the treatment of chronic and acute inflammation, infection, pain and fever. The phytochemical characterisation of the bark extract of this plant indicated that its main component is salicin, a precursor of the anti-inflammatory acetylsalicylic acid [19]. The bark extract also contains phenolic and flavonoid compounds with antioxidant activity [20,21,22]. In addition to antioxidant and anti-inflammatory properties, willow bark extract is used in weight loss supplements [23], and for its hypocholesterolemic activity [24,25]. Few data are available on the use of *Sa* in poultry species, despite acetylsalicylic acid and sodium salicylate being considered safe for poultry and used in avian medicine [26].

In this study, we evaluated the safety and the effects on performance, behaviour and blood biochemistry of the dietary supplementation with two anti-inflammatory phytoextracts, *Bs* and *Sa*, in white Leghorn laying hens reared with the rural free-range method. In particular, the effects of the two phytoextracts were studied during a critical period of the hens’ lifecycle, the transition from pre-deposition to the start of egg deposition, monitoring the production parameters and selected serum analytes. This is the first study focused on the blood biochemistry of the Leghorn breed; therefore, the data obtained can be considered an attempt to collect preliminary information on this important Italian breed.

## 2. Materials and Methods

### 2.1. Animals, Study Design and Sampling

A total of 120 17-week-old (120-day-old) pure breed white Leghorn pullets certified *Salmonella*-free, vaccinated for Newcastle Disease and Infectious Bronchitis and non-beak trimmed, were randomly assigned to 2 experimental groups, a Control group (C) and a Treated group (T), of 60 pullets each, housed in 2 single hen houses.

The trial lasted 19 weeks, from mid-August to December and was conducted at a rural farm located in Tuscany, in Mugello (43°59′20.2″ N 11°27′58.1″ E, Florence, Italy). Based on the physiological phases of the hens, the study of the performance was divided into 3 different periods, reported as weeks of observation: (*i*) first phase (1° P—7 weeks), from the supplement administration (1st week) to the start of laying (7th week); (*ii*) second phase (2° P—5 weeks), from the start of laying until the end of the supplementation (8th to 12th week); (*iii*) third phase (3° P—7 weeks) from the stop of supplementation to the end of the trial (13th to 19th week), with hens in laying phase.

Regarding the biochemical analyses, including the evaluation of H/L ratio, 4 blood samplings were performed at different timepoints: at the start of the trial (t0), after 5 weeks of supplementation (t1), at the end of supplementation (t2—12 weeks of observation) and at the end of the trial (t3—19 weeks of observation). Blood samples (2.5 mL) were collected from brachial vein using sterile syringe with 23 G-0.60 mm needle, in clean centrifuge tubes. At each experimental timepoint, the same hens were sampled. Each blood sample was divided into 2 tubes (with and without EDTA as an anticoagulant). A minimum of 0.2–0.3 mL of whole blood was kept in EDTA tubes for the evaluation of Heterophil/Lymphocytes (H/L) ratio. The serum was obtained from the remaining blood after centrifugation at 1500× *g* at 4 °C for 10 min and stored in 2 mL plastic vials at −80 °C until analysis. From the same birds, IgA were also detected in cloacal cotton swabs and in serum. The swabs were dispensed in 5 mL vials and maintained at −20 °C until analysis.

### 2.2. Management and Feeding

The 2 groups of animals were managed following the same procedures. Throughout the experiment, the natural photoperiod and temperature were maintained to permit the animals to continue their natural development until sexual maturity and laying. The average environmental temperatures were between 31.0 °C (mid-August) and 28.3 °C (September) and from 14.5–21.7 °C (October) to 5.4–6.5 °C (December). The hen houses were wooden, fenced and closed over the top with an anti-bird of prey net and located inside a wood. In each hen house, the nests for laying (1 nest/5 hens) and 20 cm/hen of perches were available. Each group was separated with a net and each hen house had an external paddock for scratching. The outside paddock consisted of an activity area without pasture or vegetation to eliminate interference with the normal feed intake. The hens had free daytime access to the paddock (from 07:00 a.m. to 16:30 p.m.) until they returned to the hen house at night.

Feeders and plastic water tanks (2 per type outdoor for each group then brought inside the hen house at night, for the first intake of feed and water in the morning) provided *ad libitum* feed and water. On rainy days, to prevent animals from drinking rainwater, they were closed inside hen houses.

For 1 week after housing, both groups received a commercial diet (*Cargill s.r.l.* feed, Table 1) gradually offered to get the animals used to the new diet and minimise diet change stress. Then the T group received a complementary feed for 12 weeks (0.3%) containing 5% of a standardised commercial dry extract of *Bs* (containing 24% of boswellic acids) and 5% of a standardised commercial dry extract of *Sa* (containing 43% of salicin). The combined use of the two extracts can be justified by the possible future use of the supplement as a commercial product due to the synergistic effect of the two phytocomplexes on the inflammatory response: *Bs* extract acts on lipoxygenase, while *Sa* extracts act on cyclooxygenase. Once the integration with phytoextracts was suspended, the feeders and water tanks were carefully washed and disinfected to avoid potential carry-over effects. From the 13th to the 19th week the T group received the commercial diet without supplementation. This period was included in the trial to observe potential long-term effects, including toxic effects, of the supplementation. The C group received the commercial diet without supplements throughout the trial. The animals were not subjected to any medical treatment plan.

### 2.3. Leghorn Hens’ Performance

The performance data were recorded during all the trial periods. At t0, for each experimental group, 8 hens were randomly selected as a sample unit and identified with numbered irremovable rings.

For each group, feed and water consumption and egg production was recorded daily, as feed intake (FI-expressed as g/hen/day), water intake (WI-expressed as mL/hen/day), obtained based on the feed and water residue left and weighed at the end of each day. The number of eggs produced was expressed as *n*° egg produce daily (*n*° eggs/day per group), drawing up a production curve with deposition rate (DR%). Instead, body weight (BW) and egg weight (*n* = 20 per group, expressed in g) were recorded weekly. Egg mass (Em) was calculated as egg-laying rate × egg weight/100 and feed efficiency (FE) as FI/Em (g/g), whereas feed conversion ratio (FCR) was calculated every week throughout the whole experimental period, from when the laying phase started.

### 2.4. Mortality and Behaviour Observations

The mortality was recorded daily throughout the experiment for each group. During the experiment, specialised technicians controlled the animals several times during the day. Attention was also paid to the establishment of the social hierarchy (pecking order) and laying behaviour. Other observations, such as the attitude to exploit the external paddock and the resourcefulness to explore the external environment, were considered.

The percentage of prolapses was also calculated for each group.

### 2.5. Health Checks

At 8, 12, and 19 weeks, 10 eggs for each group were tested for *Salmonella* spp. on shell and yolk pool separately. The microbiological test in eggs was performed according to the UNI-EN-ISO 6579-1:2017 procedures. For each group, yolk and shell pools were adequately homogenised in a stomacher. Two hundred twenty-five millilitres of Buffered Peptone Water-BPW at room temperature were added to 25 g of matrix (yolk and shell separately) and incubated at 36 °C for 18 ± 2 h. Afterward, two selective-enrichment liquid media, Rappaport-Vassilliadis Soy-RVS (Oxoid Deutschiand GmbH, Wesel, Germany) and Muller Kaufmann Tetrathionate Novobiocin-MKTTn (Oxoid Deutschiand GmbH, Wesel, Germany) were inoculated with 100 μL and 1.0 mL of culture broth, respectively, and incubated at 41.5 °C and 37 °C for 24 ± 3 h, respectively. In duplicate, a loop-full of broth was streaked on Xylose Lysine Desoxycholate-XLD (Oxoid Deutschiand GmbH, Wesel, Germany) and Brillant Green Agar-BGA (MEUS s.r.l., Piove di Sacco, Padua, Italy) and incubated at 37 °C for 24 h. Colonies referable to *Salmonella* spp., appear pink with or without a black point in the middle of the colony on XLD or BGA medium. At least once a month and for the whole period of the trial, a parasitological exam from a pool of 250 g of faeces was carried out for each group to highlight any parasitic forms.

### 2.6. Serum Biochemistry

A profile of 13 analytes was chosen, including: alanine aminotransferase (ALT), aspartate aminotransferase (AST), alkaline phosphatase (ALP), bilirubin, glucose, cholesterol, triglycerides, total proteins (TP), albumin, globulins, uric acid, calcium, phosphorus. Analyses were performed using available commercial kits (Olympus Systems Reagents), with an automated biochemical analyser (Olympus AU400, Mishima Olympus Co., Ltd., Shizuoka, Japan).

### 2.7. Serology for the Detection of Anti-Infectious Bronchitis Virus (IBV) Antibodies

Anti-IBV antibody titers were determined in serum samples using a commercial kit (IDEXX IBV Ab Test-IDEXX), following the manufacturer’s instructions.

### 2.8. Heterophils and Lymphocytes (H/L ratio) Percentages Assessment by Flow Cytometry

Fixation of blood samples was obtained by the addition of IntraPrep Reagent 1 (Beckman Coulter, Life sciences, Indianapolis, IN, USA) according to the manufacturer’s instruction (50 µL blood and 100 µL reagent 1). Blood samples were stored at +4 °C for 48 h. Two samples were obtained from each hen as a technical duplicate.

Heterophils and Lymphocytes percentage was assessed using a single staining CD45-APC (Invitrogen, Carlsbad, CA, USA), no-lyse no-wash method, as described by Seliger and colleagues [27]. Briefly, 20 µL of fixed-EDTA-blood were diluted with 980 µL of PBS 1X, then 100 µL of the diluted blood sample were mixed with 0.5 µL of CD45-APC antibody in a tube and incubated for 45 min in the dark at +4 °C. Negative controls, to evaluate inherent background or auto fluorescence, were obtained omitting primary antibodies. Subsequently, 400 µL of PBS 1X were added and samples were kept on ice and protected from light until analyses. From each sample 100 µL were analysed by MacsQUANT^®^ Analyser 10 (Miltenyi Biotec, Bergisch Gladbach, Germany). Data were analysed using the Flowlogic software (Inivai Technologies, Mentone Victoria, Australia). To start, not only debris but also artefacts (very high FSC-A) were excluded using forward (FSC-A) vs. side scatter (SSC-A). Doublets exclusion was achieved by plotting FSC-area vs. height (FSC-A/FSC-H). CD45-APC positive singlets population was gated and plotted on forward scatter (FSC) and side scatter characteristics (SSC) as described by Naghizaden and colleagues [28].

In the cytogram derived by this gating strategy, H and L area and percentage were defined, and H/L ratio was calculated.

### 2.9. IgA Determination from Cloacal Swab and Serum

The IgA extraction from cloacal swabs was performed as reported by Merino-Guzman [29]. Briefly, after thawing samples, 1 mL of PBS (Phosphate Buffered Saline) buffer containing Tween 20 (0.5%) was added to each sample without removing the swabs, vortexed for 30 s and centrifuged at 1500× *g* for 10 min at +4 °C. The supernatants were collected and analysed (dilution 1:100), together with serum sample, by a specific ELISA kit (Chicken IgA ELISA kit Cat. E33-103, Bethyl Laboratories Inc., Montgomery, TX, USA) to evaluate the IgA concentration according to the manufacturer’s instructions. Total protein concentration in cloacal samples was evaluated using Total Protein kit (Total Protein kit Micro Lowry Peterson’s Modification, Cat. TP0300, Sigma, St. Louis, MO, USA) to normalise the IgA content on protein concentration. Data are expressed as ng IgA/µg total proteins.

### 2.10. Statistical Analysis

For the evaluation of the performance, the Wilcoxon (Mann–Whitney) Rank Sum test was used to test if samples were drawn from populations with the same distributions, and the Kruskal–Wallis test (KWt) was applied to evaluate if samples were from the same population (as it is a multisampling generalisation of the two-sample Wilcoxon/Mann–Whitney Rank Sum test). The Kruskal–Wallis test was used to test the robustness of the results. The *p*-value obtained from the application of Wilcoxon (Mann–Whitney) Rank Sum test was omitted for the sake of brevity. Therefore, in Table 2, a single *p*-value derived from the application of KWt was reported because the differences obtained between the two experimental groups matched for both tests. The statistical analysis of performance data was performed for each phase (Section 2.1.). The data obtained from the Flow Cytometry tests, serology and biochemistry of blood were analysed using the Mann–Whitney test. The IgA data were analysed using the Kruskal–Wallis test. All tests were applied using STATA^®^—Statistical Software Package (StataCorp, College Station, TX, USA), version 16 (StataCorp, 2019) and the results were considered statistically significant at *p* < 0.05. The values are reported as mean ± SD (standard deviation).

## 3. Results

### 3.1. Leghorn Hens’ Performance

Feed and water consumption (FI and WI)—The data on feed and water consumption show statistically significant differences in the different phases of the study and are reported in Table 2.

In the first phase (1° P—7 weeks), during the first 7 weeks of observation, from the start of the trial to the start of laying, a significantly higher water consumption (*p* < 0.05) was measured for the T group, with mean values of 140.1 ± 35.5 mL/hen/day and 125.6 ± 38.2 mL/hen/day for the T and C group, respectively. On the other hand, the feed consumption did not show statistically significant differences between the two groups (*p* > 0.05) with mean values of 71.9 ± 18.5 g/hen/day and 70.2 ± 17.6 g/hen/day for the T and C group, respectively.

In the second phase (2° P—5 weeks), from the start of laying to the end of supplementation, which coincided with the early stage of the laying period for both groups, for water consumption, statistically significant differences were found between the groups. For the T group, the consumption of water was statistically higher compared to the C group (*p* < 0.05), with mean values of 208.5 ± 46.3 mL/hen/day (Table 2). For feed consumption, however, no significant difference was found between the groups (Table 2).

In the third phase (3° P—7 weeks) from the end of supplementation to the end of the trial, no significant differences in water and feed consumption were found. Overall, during the whole period of the trial, independent of the physiological phase of the hens, the T group had a significantly higher water consumption than the C group (*p* < 0.05), but not for feed (Table 2). For both groups, the mean daily feed consumption was 108.3 ± 33.2 g/hen/day and 105.3 ± 33.4 g/hen/day for the T and C groups, while the water consumption was 198.7 ± 61.2 mL/hen/day and 181.2 ± 67.7 mL/hen/day, respectively.

Body weight (kg-BW), FCR and FE—The BW was not significantly different, with the exception of the values recorded in the third period, between the 12th and 19th week of observation (3° P—7 weeks), where a statistically significant difference was highlighted (*p* < 0.05), with a mean value of 1.7 ± 0.0 kg-BW and 1.8 ± 0.0 kg-BW for the T and C groups, respectively. The FCR in the pre-laying phase from the 1st to the 7th week of observation was 1.7 ± 1.06 and 1.2 ± 0.40 for T and C, respectively, and not statistically different (Table 2). From the 8th to the 19th week of the trial, the hens were in the laying phase and FE recorded mean values of 2.8 ± 0.7 g/g for the T group and 3.3 ± 1.7 g/g for the C group, but these were not statistically different (*p* > 0.05) (Table 2).

**Figure 1:**
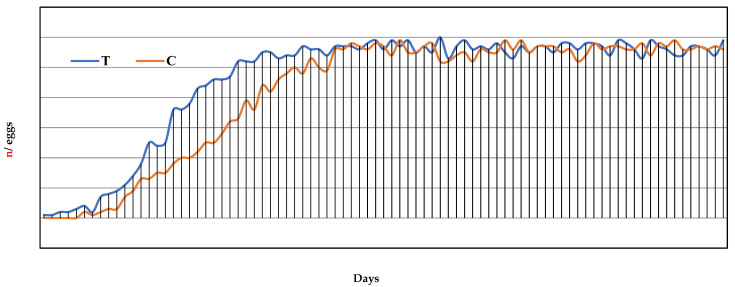
Egg production curve for a total of 12 weeks of laying, corresponding to the 7th to 19th week of the trial, expressed as day of laying. T = supplemented hens (*n* = 60); C = control hens (*n* = 60).

Egg production—The egg production curve is reported in Figure 1. The egg-laying started in the 8th week of the trial for the T group and 6 days later for the C group (Table 2). It can be noted that the T group, already showed an increase in weekly eggs laid from the 1st week and a relative DR% greater than the C group (Figure 1). Between the 13th and 14th week of observation, corresponding to the 5th and 6th week of laying, the T group reached its productive peak with a DR% between 95% and 98.3%, while the C group reached its highest rate only at the 7th week of laying, with a DR% of 98.3%. From the 6th week to the 12th week of laying, a >95% weekly DR% was recorded, and from the 1st to 12th week of laying there was a regular increase in egg mass (Em) equal to 50.1 ± 10.7 and 46.3 ± 15.07 for the T and C groups, respectively (Table 2). However, for Em and FE, no statistically significant differences were found between the groups (*p* > 0.05). The daily mean number of eggs produced per group was 46.6 ± 17.9 and 44.4 ± 18.0 for the T and C groups, respectively, while the total number of eggs produced was 3924 vs. 3559 for the T and C groups, respectively. Significant differences were detected in the egg weight. In fact, the weight of the eggs produced by the T group was statistically lower than the weight of the eggs of the C group (*p* < 0.05), with mean egg weight values of 59.9 ± 2.5 g and 61.0 ± 3.4 g, respectively. No cases of oviduct prolapse were detected in both groups.

### 3.2. Mortality, Behaviour Observations and Health Checks

Throughout the trial, no mortality and cases of pica and cannibalism were recorded. In the T group, no abnormal behaviour was observed in feed intake, despite the perceptible and typical smell of phytoextracts. However, after 12 days of supplementation, a change in behaviour was observed in the hens of the T group, characterised by drowsiness, reduced reactivity and reduced restlessness. The peaks of inactivity were characterised by stopping on the perch or on the litter, in a position with the head under the wing, which was found at consistent times from approximately 9.30 a.m. to 15.00 p.m. However, this behaviour did not affect FI and WI (Table 2). The behaviour was maintained until the integration was stopped. Six days after the suspension of the supplement, the hens assumed the typical normal behaviour of the breed. In the C group, no abnormal behaviour occurred, and the FI and WI were constant. Both groups were monitored once a month for the presence of intestinal parasitic forms, such as Salmonella spp. infection, always resulting in a negative and with an isolation rate of 0.00%.

### 3.3. Serum Biochemistry

The effect of dietary supplementation on selected serum analytes is reported in Table 3. No significant differences were determined for the analysed serum analytes between the T and C groups, with few exceptions. Albumin at t0 and t2 was significantly higher (*p* < 0.05) in the C group with respect to the T group, ALT at t3 was significantly higher (*p* < 0.05) in the T group than in the C group, and glucose at t0 was significantly higher (*p* < 0.05) in the T group than in the C group. On the other hand, significant differences (*p* < 0.05) were determined over time (t0 vs. t1 vs. t2 vs. t3) in both groups due to physiological development and laying activity: a significant decrease in hepatic enzyme activity as AST and ALP, also glucose concentration and a significant increase in triglycerides, total proteins, albumin, and Ca concentrations were observed. The analysis of these differences is out of the scope of the present research.

### 3.4. Anti-IBV Antibody Titers

**Figure 2:**
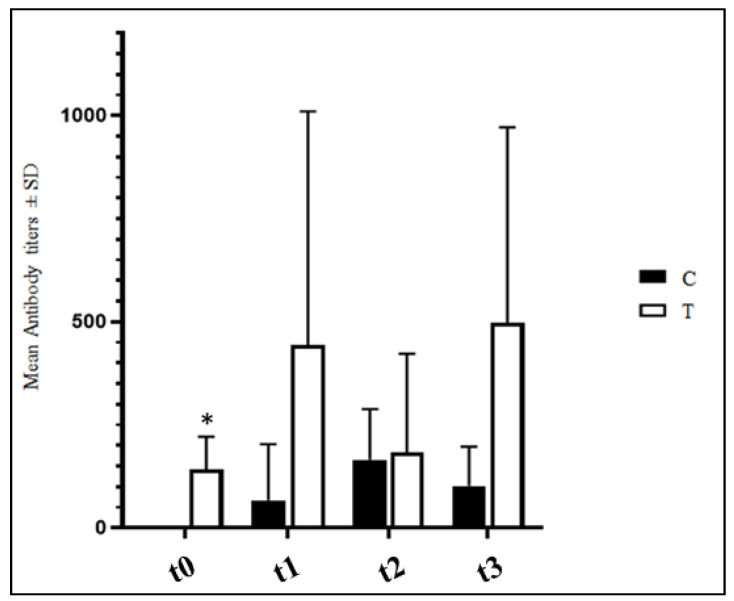
Mean IBV antibody titers ± SD detected in C and T groups. T = supplemented hens (*n* = 60); C = control hens (*n* = 60); t0 = start of the trial; t1 = after 5 weeks of supplementation; t2 = end of supplementation; t3 = end of the trial. *: statistically significant at *p* < 0.05.

ELISA anti-IBV antibody titers detected in four samples per experimental group are reported in Figure 2. No significant differences were observed between the C and T groups (*p* > 0.05) with the sole exception of t0 when the hens of the T group presented significantly higher anti-IBV antibody titers (*p* < 0.05).

### 3.5. Heterophils and Lymphocytes (H/L) Ratio

**Figure 3:**
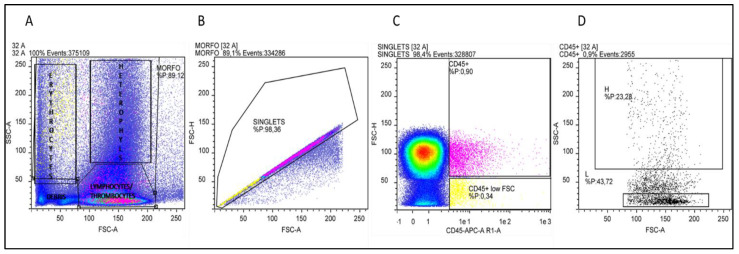
Results of gating strategy in Flow Cytometry analysis. (**A**) Representative cytogram (FSC-A versus SSC-A): debris is located at the lower-left corner of the cytogram, nucleated erythrocytes appear as a smear on the left, in the lower-right area lymphocytes and thrombocytes are placed, in the upper-right area heterophils are contained. (**B**) FSC-A/FSC-H plot for doublets exclusion: singlets have a proportion between area and height values while doublets have double the area values of single cells whilst the height is the same. (**C**) Singlets were analysed for CD45-APC fluorescence intensity. In the area with the highest APC fluorescence, two populations can be identified: a population with low SSC values (yellow) and a population with higher SSC values (pink) (**D**) CD45-positive cells cytogram in which lymphocytes and heterophils areas are selected.

**Figure 4:**
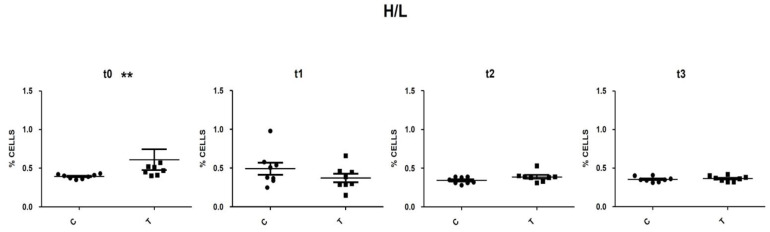
Scatter plot of H/L ratio in C and T group at t0, t1, t2 and t3. **: statistically significant (*p* < 0.01). T = supplemented hens (*n* = 60); C = control hens (*n* = 60); H/L = heterophils/ lymphocytes ratio; t0 = start of the trial; t1 = after 5 weeks of supplementation; t2 = end of supplementation; t3 = end of the trial.

Cellular debris located at the lower-left corner of the cytogram (forward versus side scatter plot) was excluded from the analysis. Doublets have double the area values of single cells whilst the height is roughly the same and so disproportions between height and area were used to eliminate doublets. The singlets’ population was evaluated for the expression of the CD45 pan-leucocytes antigen. It is important to point out that the nucleated erythrocytes appeared as a smear on the left and, as reported by Seliger and co-workers [27] on whole blood staining, the autofluorescence of erythrocytes leads to a relatively high background fluorescence of the CD45 negative population, which overlaid the signal of the CD45 low thrombocytes (Figure 3). By selecting the population with the highest APC fluorescence values and excluding, in this fluorescence range, the population with the lowest SSC values (low complexity cells) thrombocytes could be excluded. The positively selective population was evaluated based on the morphological parameters and lymphocytes (low FSC and SSC) and heterophils (high FSC and SSC) areas were identified (Figure 3). This gating strategy was applied to all samples analysed by Flow Cytometry, heterophils and lymphocytes percentages and the H/L ratios were calculated. At t0, control animals had an H/L ratio value of 0.4. No significant differences were detected between the T and C groups, with the sole exception of t0, when the T group presented an H/L ratio value significantly higher than that of the C group (*p* < 0.05) (Figure 4). At t1, the H/L ratio of the T group significantly decreased (*p* < 0.05) returning to control values (≤ 0.4).

### 3.6. IgA Concentration in Faecal Swabs and Serum Samples

**Figure 5:**
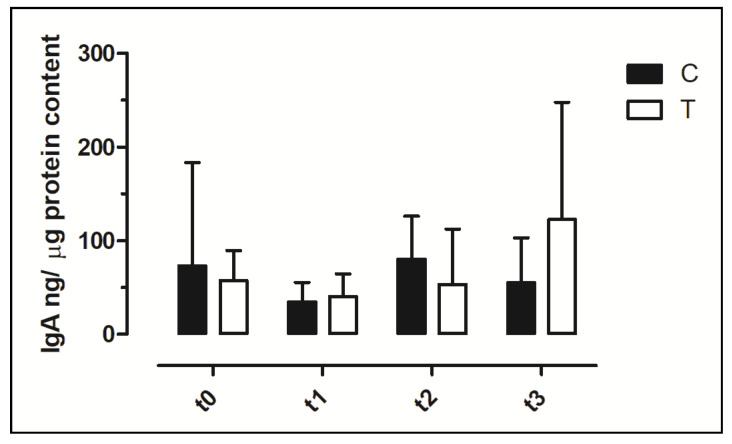
Concentration of Immunoglobulin A (IgA) in faecal swabs. The data are reported as mean ± SD. T = supplemented hens (*n* = 60); C = control hens (*n* = 60); t0 = start of the trial; t1 = after 5 weeks of supplementation; t2 = end of supplementation; t3 = end of the trial.

In all samples, it was possible to detect the IgA concentration. The IgA faecal content showed a wide variability among animals and among timepoints (Figure 5). No significant differences were observed in faecal IgA between the C and T groups and during the timepoints (*p* > 0.05).

## 4. Discussion 

Investigations carried out on a variety of domestic animals have confirmed the multiple effects of phytoextracts on nutrition, health improvement and productivity [5,14,32,33]. However, studies on the use of *Sa* and *Bs* extracts in poultry are scarce or even lacking in laying hens. This research can be considered the first attempt focused on the effects of the combination of the two phytoextracts in free-range Leghorn hens.

Leghorn Hen Performance and Behaviour—This study indicated a significant effect of supplementation on performance. Significant differences were found between the two experimental groups in the consumption of water, body weight and egg weight. These differences were evident particularly during the second period of the trial when supplementation overlapped with the start of laying. Hens from the T group showed significantly higher consumption of water and started the laying phase 6 days before the hens of the C group. During the laying phase, a greater consumption of water is considered normal, but nevertheless, the hens of the T group consumed more water compared to the C group. The presence of additional NaCl in the complementary feed, though in a very low percentage (0.35%), might have contributed to the increased water consumption in the T group. The production peak and the deposition rate of 100% were reached faster by the supplemented hens, despite the fact that they demonstrated slowed development of their secondary reproductive somatic characteristics (crest and wattle development) 15 days following the administration of the supplement. This observation in hens supplemented with *Bs* and *Sa* is not documented by other studies and remains unexplained.

The results of the present research indicated that the supplementation of *Bs* and *Sa* in the diet significantly improved egg production during the first 5 weeks of laying, and hens from the T group produced a higher number of eggs than those of the control group. Significant improvement in egg production was also reported in hens that were fed diets supplemented with pre/pro-synbiotics [31]. However, at the end of the trial, the eggs of the T group had a significantly lower weight than those of control hens. 

In the 3° P, the BW was significantly lower for hens of the T group, possibly due to the effects of the supplement. Data on the effect of *Bs* extract on BW are not available in the literature, while willow bark extract is used in weight loss products for humans, frequently in association with other phytoextracts, such as extracts of green tea and ginger. Accordingly, Pozniak and a co-worker found that the BW gain decreased in broilers when the feed was supplemented with 0.04% of acetylsalicylic acid; this effect is presumably related to anti-inflammatory and analgesic activities of the phytocomplex [26]. However, a paucity of controlled clinical trials has been performed to evaluate the efficacy of willow bark extract and the use in association with other extracts hampers the evaluation of its direct efficacy on weight loss [23].

The behavioural changes observed in the hens of the T group are interesting; in fact, the psychoactivity of *Boswellia* extract has been recognised since ancient times. Accordingly, sedative and hypnotic effects were recently reported in mice treated with silver nanoparticles loaded with AKBA, one of the most active terpenes isolated from the oleogum resin of different species of the genus *Boswellia* [34]. Moreover, Moussaieff and co-workers showed that incensole acetate, a diterpene present in *Boswellia* resin, is an agonist of the ion channel transient receptor potential vanilloid (TRPV) [35]. The activation of the channel determined anxiolytic-like and antidepressive-like behavioural effects in mice. Finally, Okano and colleagues reported that *Boswellia* essential oil counteracts the negative effects of stress by effectively relieving sleep debt in rats [36]. The increased drowsiness and the reduced reactivity observed in the hens of the T group during the supplementation might be due to the combined effects of the aforementioned active molecules and add additional in vivo evidence of the sedative effects of *Bs* extracts.

Blood Biochemistry, H/L ratio and Faecal IgA—To the author’s knowledge, data on blood biochemistry in Leghorn pure breed are limited to AST, ALT, uric acid and creatinine [37]; therefore, the present results will be discussed in the framework of knowledge also obtained in other chicken breeds. Recently, Board and co-workers [30] reported the biochemical reference intervals for backyard hens (Table 3). The data obtained in this study fall in those reference intervals with the sole exception of glucose at t1 in the T group, which is higher than the upper limit reported by Board and colleagues [30], but it falls in the reference interval reported for Lohman silver laying hens by Ding and co-workers [38]. The activity of enzymes in the serum provides important information on the integrity and functionality of specific organs. In birds, the activity of serum AST is considered the most reliable biomarker of hepatic function [37]. However, serum AST can also originate from muscles; consequently, in this study, two other enzyme biomarkers of hepatic function, namely ALT and ALP, were also analysed. The supplementation did not negatively affect the activity of these enzymes, suggesting the absence of hepatotoxicity. Similar results were reported in Leghorn hens supplemented with silkworm pupae [39], and in Lohmann LSL-Lite and Lohmann Brown-Lite laying hens supplemented with *Chondrus crispus* and *Ascophyllum nodosum* [40]. In birds, uric acid, the major end product of nitrogen catabolism, is excreted by the kidneys and is considered a biomarker of renal function. The data obtained in this study fall within the interval reported for laying hens [30] and no significant variations were detected between the C and T groups, suggesting the absence of impairment of renal function. Despite the well-recognised hypoglycaemic and hypocholesterolemic effect of *Bs* extracts in mammals [41], in the present study, no significant reduction of glucose and cholesterol was observed in the serum of hens fed the supplemented diet. Accordingly, no effect of *Bs* on the serum lipid profile was noted by Kiczorowska and co-workers [18] in broilers, and the concentration of serum cholesterol did not show significant differences between broilers fed a control diet and broilers fed diets supplemented with 0.025% and 0.05% *Sa* extracts [42]. Regarding glucose, no significant differences were reported in broilers fed diets supplemented with 3, 4, and 5% *Bs* [5], whereas a significant decrease in serum glucose was observed in broilers fed diets supplemented with 0.025% and 0.05% *Sa* [42]. To the author’s knowledge, no studies are reported for laying hens.

The H/L ratio has become widely accepted as an indicator of stress responses in poultry [43,44] since Gross and Siegel [45] first found decreasing numbers of lymphocytes and increasing number of heterophils in response to different physiological stressors. Gross and Siegel concluded that the H/L ratio was a more reliable parameter than plasma corticosteroids for the measurement of stress in poultry and that it was also less variable than total cell numbers. The use of haematology analysers in mammalian veterinary medicine has led to a considerable reduction of labour costs with more reliable results compared to traditional microscopic procedures. Haematological peculiarities of birds, in particular nucleated erythrocytes and thrombocytes, have precluded the successful analysis of avian blood samples by current haematological analysers [27,46]. Flow Cytometry protocol utilised in this study proved to be effective as the H/L values obtained are comparable to those reported in the literature for healthy non-stressed chickens [47]. The random difference existing between the two groups at t0 showed a higher H/L ratio in the T than in the C group, with a value higher than the indicative value of 0.4 reported in the literature for non-stressed birds [45,48]. At t1, following the supplementation, in the hens from the T group, the H/L ratio significantly decreased, returning to values < 0.40. These observed changes could be suggestive of a potential beneficial effect from the *Bs* and *Sa* extracts. 

Three classes of immunoglobulins (IgY, IgA and IgM) are expressed in chickens, and IgA play an essential role in mucosal defense [49]. As IgA are the major antibody component of the gastrointestinal mucosa, the IgA content in the faeces can be considered a marker of the local immune system and an increase could be due to enhanced protection against pathogens. However, increased IgA concentrations might also represent a response to increased antigenic stimulation without enhanced immunity or to the body’s protective mechanism against a harmful stimulus [50]. Different papers reported the possibility to quantify IgA in the intestinal lumen [51], faecal samples [52] and cloacal swabs [29]. The concentrations of IgA measured in this research showed wide variations among animals and timepoints with a mean value at t0, if expressed in ng/mL, 10 times higher than the value reported in the study by Merino-Guzman [29]. The difference may be due to the different management of animals and the diet utilised. Due to the difference in cloacal swab sampling, in terms of the amount of faeces present on the swab, we decided to quantify the protein content of each sample and express the IgA data as ng/µg protein. As faecal IgA concentration was used to evaluate the effect of different dietary supplementation on mucosal defense in poultry [52,53], we investigated the IgA content in cloacal samplings and no significant change after dietary supplementation was observed.

## 5. Conclusions

Overall, the supplementation with *Bs* and *Sa* resulted in a significant decrease in egg weight accompanied by a reduction of BW. In addition, reduced activity occurred during the supplementation, suggesting a possible sedative effect of the supplement. The authors are aware that the use of a supplement containing two different phytoextracts has made the discussion of the results particularly complex because it was not possible to determine the specific contribution of the single extract. However, in the author’s opinion, it is important to test the efficacy and safety of supplements that often contain mixtures of different phytoextracts. Nonetheless, the supplementation was safe, did not compromise the productivity and the performance of the treated group and maintained the welfare status. Finally, it should be evidenced that hens reared with a free-range method have a potentially better welfare status compared to the hens reared with traditional intensive methods. In the future, therefore, it would be interesting to also test this supplement in laying hens reared on intensive farms.

## Figures and Tables

**Table 1:** *Cargill s.r.l.* feed formulation based on only the indications of the commercial tag and complementary feed composition.

Composition	Values of Nutrients (%/kg of Finisher Diet)	Additives (mg/kg; IU; OTU/kg)	Complementary Feed Composition (%)
Corn; Corn gluten flour; Soybeans meal (* CP 43%); Calcium carbonate; roasted Soybeans; Rice husk; Corn gluten; Wheat bran; Soybean oil	CP, 17%; ** CF, 5%; *** CF, 3.51%; ^!^ Cash, 13.27%; ^ǂ^ Ca, 4.02%; ^ǂǂ^ P, 0.58%; ^≠^ NaCl, 0.15%; ^+^ Ly, 0.85%; ^$^ Met: 0.33%.	Vitamin A, 9950 IU; Vitamin D_3_, 2701 IU; Vitamin E, 38 mg; Vitamin K3, 2 mg; Vitamin B1, 1.5 mg; Vitamin B2, 4.5 mg; Vitamin B6, 2.5 mg; Vitamin B12, 0.008 mg; niacin, 35 mg; Ca-D-pantotenate, 10 mg; folic acid, 1 mg; biotin, 0.1 mg; betaine hydrochloride, 250 mg; Cu, 5 mg; anhydrous calcium iodate, 0.50 mg; Mn, 50 mg; Se, 0.075 mg; Zn, 40 mg; Cantaxantine, 2 mg; promoters of digestion 6-phytase, 213 OTU; DL-Methionine, 627 mg	Calcium carbonate, 74.5%; Colloidal silica, 15%; *Salix* ^&^ DE, 5%; *Boswellia* ^&^ DE, 5%; Sodium chloride, 0.35%; Magnesium carbonate, 0.15%

* CP: Crude Protein; ** CF: Crude Fats; *** CF: Crude Fibre; ^!^ Cash: Crude ash; ^ǂ^ Ca: Calcium; ^ǂǂ^ P: Phosphorus; ^≠^ NaCl: Sodium chloride; ^+^ Ly: Lysine; ^$^ Met: Methionine; ^&^ DE: Dry Extract.

**Table 2:** Summary of the evaluations of the performance parameters.

Parameters	T	C	*p*-Value (KWt)
	Mean ± DS	Mean ± DS	
Kg/BW 1°P (1–7 week)	1.0 ± 0.1	1.1 ± 0.1	*p* > 0.749
Kg/BW 2°P (8–12 week)	1.5 ± 0.1	1.5 ± 0.1	*p* > 0.601
Kg/BW 3°P (13–19 week) ”	1.7 ± 0.0	1.8 ± 0.0	* *p* < 0.004
FI 1°P (1–7 week) **	71.9 ± 18.5	70.2 ± 17.6	*p* > 0.100
FI 2°P (8–12 week)	115.8 ± 16.8	109.5 ± 16.9	*p* > 0.077
FI 3°P (13–19 week)	139.8 ± 8.3	139.4 ± 8.9	*p* > 0.970
FI (1–19 week)	108.3 ± 33.2	105.3 ± 33.4	*p* > 0.516
WI 1°P (1–7 week) ***	140.1 ± 35.5	125.6 ± 38.2	* *p* < 0.013
WI 2°P (8–12 week)	208.5 ± 46.3	163.0 ± 46.6	* *p* < 0.000
WI 3°P (13–19 week)	251.4 ± 32.0	251.2 ± 34.1	*p* > 0.929
WI (1–19 week)	198.7 ± 61.2	181.2 ± 67.7	* *p* < 0.013
FCR (1–7 week) ^!^	1.7 ± 1.0	1.2 ± 0.4	*p* > 0.609
Feed Efficiency (8–19 week) ^£^	2.8 ± 0.7	3.3 ± 1.7	*p* > 0.508
Egg mass (2–12 week/laying) ^#^	50.1 ± 10.7	46.3 ± 15.0	*p* > 0.921
*n*° eggs/day/lay (1–12 week/laying)	46.6 ± 17.9	44.4 ± 18.0	*p* > 0.246
*n*° total egg/group (1–12 week/laying)	3924	3559	*p* > 0.068
Mean DR% (2–12 week/laying) ^$^	83.4 ± 26.6	75.3 ± 31.8	*p* > 0.418
Egg weigth (*n* = 20/group) (3–12 week/laying) ^&^	59.9 ± 2.5	61.0 ± 3.4	* *p* < 0.000

^”^: body weight expressed as kg/BW; **: Feed Intake, expressed as g/day/hen; ***: Water Intake, expressed as (mL/day/hen); ^!^: Feed Convertion Ratio; ^£^: Feed Efficiency = Feed Intake/Egg mass (g/g); ^#^: Egg mass = (egg production × egg weight)/100; ^$^: Deposition rate (DR%); ^&^: weight expressed in g; *: statistically significant at *p* < 0.05.

**Table 3:** Serum biochemical analytes in Leghorn hens. Data are reported as mean ± SD (*n* = 8 for each group and at each. Timepoint: at the start of the trial (t0), after 5 weeks of supplementation (t1), at the end of supplementation (t2—12 weeks) and at the end of the trial (t3—19 weeks).

Serum Analytes	t0		t1		t2		t3		From Reference ^§^
	C	T	C	T	C	T	C	T	
AST (U/L)	270 ± 40.0	243 ± 21.94	216 ± 20.4	207 ± 12.5	221 ± 25.8	207 ± 17.7	214 ± 16.3	217 ± 25.1	118–298 [30]
ALT (U/L)	1.25 ± 0.46	1.71 ± 0.76	1.88 ± 0.64	2.50 ± 0.93	1.57 ± 0.53	1.38 ± 0.52	1.63 ± 0.74	2.88 ± 1.25	25.2 ± 7.77 [31]
ALP (U/L)	1159 ± 310	1510 ± 367	713 ± 182	673 ± 98.5	730 ± 167	595 ± 209	480 ± 206	552 ± 236	407 ± 39.84 [31]
Bilirubin (μmol/L)	0.17 ± 0.00	0.34 ± 0.17	0.88 ± 0.68	0.34 ± 0.17	0.34 ± 0.32	0.34 ± 0.17	0.51 ± 0.34	0.51 ± 0.51	--
Cholesterol (mmol/L)	3.42 ± 0.17	3.16 ± 0.35	2.98 ± 0.17	2.92 ± 0.31	3.19 ± 0.55	3.00 ± 0.71	2.98 ± 0.74	3.32 ± 0.62	3.37 ± 0.35 [31]
Triglycerides (g/L)	1.05 ± 0.49	0.90 ± 0.21	1.29 ± 0.19	1.15 ± 0.10	12.6 ± 3.38	12.6 ± 4.63	12.29 ± 5.94	17.36 ± 3.46	13.6 ± 3.56 [31]
Glucose (mmol/L)	12.9 ± 1.23 *	14.3 ± 0.74 *	8.63 ± 0.79	9.35 ± 1.21	10.78 ± 0.49	11.2 ± 1.32	7.20 ± 1.72	7.70 ± 1.96	9.41–13.6 [30]
Total proteins (g/L)	42.7 ± 3.13	39.3 ± 2.92	48.4 ± 3.62	46.2 ± 4.11	48.7 ± 4.85	46.8 ± 4.29	45.9 ± 3.40	48.3 ± 4.09	39–70 [30]
Albumin (g/L)	16.3 ± 1.03 *	15.0 ± 0.80 *	19.2 ± 1.19	18.4 ± 1.46	18.7 ± 1.10 *	17.5 ± 0.75 *	18.3 ± 0.82	18.8 ± 0.84	15.0–33.0 [30]
Globulins (g/L)	25.8 ± 1.85	26.2 ± 4.92	29.2 ± 2.58	27.8 ± 3.17	30.0 ± 4.01	29.2 ± 4.15	27.7 ± 2.70	29.5 ± 3.34	16.0–43.0 [30]
Albumin/ Globulins	0.65 ± 0.06	0.61 ± 0.05	0.66 ± 0.03	0.67 ± 0.07	0.63 ± 0.06	0.61 ± 0.07	0.67 ± 0.05	0.64 ± 0.05	--
P (mmol/L)	1.78 ± 0.32	1.53 ± 0.19	2.01 ± 0.18	1.92 ± 0.23	2.01 ± 0.17	2.07 ± 0.21	2.17 ± 0.37	2.33± 0.42	0.52- 2.33 [30]
Ca (mmol/L)	2.65 ± 0.34	2.98 ± 0.20	2.70 ± 0.21	2.46 ± 0.43	6.10 ± 0.41	6.40 ± 0.63	6.75 ± 0.10	7.25 ± 0.64	> 2.73 [30]
Uric acid (mmol/L)	0.30 ± 0.06	0.28 ± 0.06	0.42 ± 0.08	0.39 ± 0.08	0.37 ± 0.11	0.33 ± 0.09	0.39 ± 0.12	0.30 ± 0.12	0.05–0.53 [30]
IgA (g/L)	0.72 ± 0.22	0.83 ± 0.09	0.70 ± 0.01	0.71 ± 0.08	0.70 ± 0.00	0.65 ± 0.03	0.63 ± 0.00	0.51 ± 0.25	--

*: statistically significant at *p* < 0.05.; ^§^:- when data in the reference were not expressed in SI unit, they were converted to SI unit.

## Data Availability

Not applicable.
